# Regulatory roles of RNA binding proteins in the Hippo pathway

**DOI:** 10.1038/s41420-025-02316-z

**Published:** 2025-01-31

**Authors:** Shuchang Peng, Chenglin Li, Yanwen He, Lei Xue, Xiaowei Guo

**Affiliations:** 1 The Key Laboratory of Model Animals and Stem Cell Biology in Hunan Province, School of Basic Medical Sciences, Hunan Normal University, Changsha, China; 2 The Engineering Research Center of Reproduction and Translational Medicine of Hunan Province, Changsha, China; 3 Department of Nuclear Medicine, Shanghai 10th People’s Hospital, School of Life Science and Technology, Tongji University, Shanghai, China; 4 Changsha Stomatological Hospital, Hunan University of Chinese Medicine, Changsha, China

**Keywords:** Cell signalling, Molecular biology

## Abstract

The Hippo pathway represents a highly conserved evolutionary pathway, dysfunction of which has been implicated in various diseases. RNA-binding proteins (RBPs) intricately modulate gene expression through interacting with non-coding RNAs or other proteins. To data, while an array of RBPs have been identified as modulators of the Hippo pathway, there remains a notable absence of a comprehensive review addressing the mechanistic regulations of RBPs in the transduction of Hippo signaling. Herein, this review aims to consolidate recent advances and elucidate the intricate mechanisms underlying RBPs binding to target RNA. It also explores the dynamic interplay between RBPs, non-coding RNAs, TFs, and DNA on chromatin. Additionally, it also outlines future perspectives, including the essential non-canonical functions of RBPs and emerging roles of non-canonical RBPs as transcription factors (TFs) in genes transcription. Overall, this review provides mechanistic insights into the roles of eukaryotic RBP proteins in the regulation of crucial signaling cascades.

## Facts

- Dysfunction of the Hippo signaling pathway has been closely associated with various diseases including cancer.
- A multitude of RNA-binding proteins have been identified as regulators of the Hippo pathway through multiple canonical mechanisms including alternative splicing, stability, and translation.
- RNA-binding proteins, Non-coding RNAs and TFs collaborate to regulate gene expression.
- Modifications of RNA binding proteins in response to different stimuli affect the Hippo pathway.

## Open Questions

- Can RNA-binding proteins regulate gene expression via non-canonical mechanisms independent of RNA-binding activity?
- Can a fraction of transcription factors function as RNA-binding proteins under specific conditions?
- Can RNA-binding proteins regulate the Hippo pathway in a feedback loop?

## Introduction

The Hippo signaling pathway, initially discovered in *Drosophila*, has been well-characterized as a conserved pathway that regulates organ size and tumorigenesis [1–6]. A myriad of upstream signals, such as cell polarity, mechanical cues, cell density and stress stimuli [3, 7, 8], have been extensively demonstrated to modulate Hippo signaling across biological processes [9–11]. In brief, the core of Hippo pathway comprises a kinase cascade that the kinase Hippo in *Drosophila* (MST1/2 in mammals) phosphorylates the downstream kinase Warts (LATS1/2 in mammals), which in turn leads to the phosphorylation and inactivation of oncoprotein Yorkie (YAP/TAZ in mammals). Disruption of the Hippo pathway results in nuclear translocation of unphosphorylated Yki/YAP/TAZ. Subsequently, these proteins synergistically cooperate with Scalloped (Sd)/TEAD1-4 (Sd orthologs in mammals) to upregulate the expression of downstream target genes [10–13].

Deregulation of the Hippo pathway has been deeply implicated in the development and various diseases. On one hand, extensive research has robustly established the oncogenic function of YAP/Yki, a fact that has been even succinctly featured in a collection of review articles. On the other hand, a growing number of studies have also bolstered the notion of the inhibitory roles of YAP/Yki. For instance, the loss of Yki/YAP markedly enhances cell migration in both *Drosophila* and human cancer cell lines [14]. Oxidative stress reprograms Yki to switch from a pro-proliferative factor to an anti-proliferative agent, establishing a self-protective mechanism [15]. In addition, it has been documented that the activation of YAP inhibits tumor growth in clear cell renal cell carcinoma, as well as in prostate and estrogen receptor α-positive breast cancers [16–18]. To sum up, the Hippo-YAP pathway could execute either tumor-promoting or suppressive functions in a context-dependent manner.

Numerous regulators of the Hippo signaling pathway have been identified successfully, primarily falling into the category of post-translational modification (PTM) enzymes, such as kinases, phosphatases, E3 ubiquitin ligases, methyltransferases, and deubiquitinating enzymes [13, 19]. Besides, a multitude of RNA binding proteins (RBPs) have also been established as key regulators of the Hippo pathway. In brief, regulatory mechanisms of Hippo pathway by RBPs are typically associated with multiple RNA processing events, such as alternative splicing, mRNA stability, translation, and nuclear localization [20]. Mounting evidence links the dysregulation of RBPs to the intricacies of normal development and a spectrum of diseases [21–25], highlighting an intriguing interplay where RBPs and non-coding RNAs collaborate or compete to modulate gene expression [19]. Mechanistically, RBPs can enhance or counteract the interaction between miRNAs and target RNAs through mediating RNA structural dynamics changes, or affecting miRNA access to the overlapping or proximal binding sites in RNA, and vice versa [26, 27].

About the regulation of Hippo pathway by RBPs, it should the first report that heterogeneous nuclear ribonucleoprotein Hrb27C could regulate Yki phosphorylation in *Drosophila* via a yet unknown mechanism [22]. In addition, our previous study provided compelling evidences that Rox8 recruits and stabilizes miRNA-8 to induce *yki* mRNA degradation, a mechanism highly conserved from *Drosophila* to humans [19]. However, there remains a dearth of comprehensive reviews addressing the intricate interplay between the Hippo pathway and RBPs. Furthermore, this review also outlines perspectives on the future research concerning the functions of RBPs, aiming to enhance the understanding of their roles in the regulation of crucial signaling cascades.

## Mechanistic regulation of the Hippo pathway by RBPs

A number of RBPs have been successfully identified as regulators of the Hippo pathway via multiple canonical mechanisms including mRNA stability, translation and alternative splicing. These regulatory processes often result in the dysregulation of cellular homeostasis during development and are implicated in a spectrum of diseases, including cancer, which were summarized as follows and listed in the Table 1.

**Table 1:** Regulation of the Hippo pathway by RBPs.

Mechanistic Basis	RBP	Putative Target	Cancer Type	Reference
**mRNA Stability**	Dnd1	LATS2	hepatocellular carcinoma (HCC)	[28]
	FUS	LATS1/2	HCC	[29]
	RNPC1	MST1/2	—	[30]
	METTL3	FZD10; LATS1	HCC; breast cancer	[34, 38]
	YTHDF2	FZD10; LATS1; YAP	HCC; breast cancer; lung cancer	[34, 38, 39]
	IGF2BP1	SIK2	Non-small cell lung cancer (NSCLC)	[35]
	Rox8/TIAR	yki /YAP	lung cancer	[19]
	LIN28	MST1/2; LATS1/2	triple-negative breast cancer (TNBC)	[36]
	MSI2	MST1/2; LATS1/2; SAV1; MOB1	TNBC; pancreatic ductal adenocarcinoma (PDAC)	[36, 41]
	QKI5	TAZ	ovarian cancer	[37]
	ALKBH5	YAP	NSCLC	[39]
	MEX3A	KIBRA	HCC	[40]
	hnRNPF	YAP	—	[42]
**Translation**	YTHDF1	YAP	NSCLC	[39]
	ALKBH5	YAP	NSCLC	[39]
	MSI2	SAV1; MOB1	PDAC	[41]
**Alternative Splicing**	ESRP2	NF2	Non-alcoholic fatty liver disease (NAFLD)	[43]
	B52	yki	—	[44]
	RBFOX2	TEAD1	—	[45]

This table summarizes the list of RBPs associated with the Hippo signaling pathway, including the mechanisms, putative targets, cancer types and selected references.

### mRNA stability

It’s a well-known notion that RBPs could increase the stability of target mRNA transcripts [20]. For instance, ectopic expression of Dnd1 promotes *LATS2* mRNA decay through binding to the *LATS2 3’-UTR*, thus promoting YAP phosphorylation and inhibiting Epithel-to-Mesenchymal Transformation (EMT) in hepatocellular carcinoma (HCC) [28]. Similarly, enforced expression of FUS amplifies LATS1/2 expression to attenuate HCC progression by stabilizing *LATS1/2* mRNA [29]. RNPC1 mitigates dryness in endothelial cells by enhancing *MST1/2* mRNA stability [30]. It is noteworthy that an increasing number of studies have highlighted the dynamic and reversible m6A methylation process, which is an essential modification of eukaryotic RNAs involving RBP methyltransferases (Writers), demethylases (Erasers), and m6A-binding proteins (Readers) [31, 32]. In brief, typical writers consist of METTL3, METTL14 and WTAP, and the main erasers are FTO and ALKBH5, while YTHDF1-3 acts as m6A readers [33]. For example, *FZD10* mRNA methylated by METTL3 is recognized by YTHDF2, thereby maintaining its stability in liver cancer cells [34]. Insulin-like growth factor 2 mRNA-binding protein 1 (IGF2BP1) stabilized *SIK2* mRNA through m6A modification to promote non-small cell lung cancer (NSCLC) progression [35]. As such, these studies demonstrate that RBPs could decrease mRNA stability.

In addition, under certain conditions, RBPs could promote mRNA decay [20]. For instance, our previous research has demonstrated that Rox8 recruits and stabilizes miRNA-8-RISCs binding to the 3’UTR of *yki* mRNA, thereby leading to *yki* mRNA degradation [19]. Two RBPs, LIN28 and MSI2, cooperate to induce the mRNA decay of YAP1 upstream kinases, including MST1/2 and LATS1/2, consequently inhibiting the Hippo pathway in triple-negative breast cancer (TNBC) [36]. QKI5 directly binds and decreases *TAZ* mRNA stability in ovarian cancer progression [37]. YTHDF2 triggers *LATS1* mRNA degradation by recognizing the m6A site of its mRNA induced by METTL3 in breast cancer cells [38]. Likewise, YTHDF2 facilitates *YAP* mRNA decay via the RISC-AGO2 system, which could be regulated by m6A modification by ALKBH5 in lung cancer [39]. In addition, MEX3A degrades *KIBRA* mRNA through a yet unknown mechanism to regulate Hippo pathway-dependent HCC [40]. MSI2 modulates pancreatic cancer development through directly binding to the mRNAs of *SAV1* and *MOB1*, thereby controlling the translation and stability of *SAV1*, as well as the translation of MOB1 [41]. The binding of hnRNPF to the *YAP 3’UTR* directly results in *YAP mRNA* degradation [42]. In summary, RBPs could regulate gene expression through altering target mRNA stability, which has been well-recognized as a canonical mechanism of RBPs.

### Translation

It has been unraveled that RBPs play indispensable roles in the intricate process of mRNA translation [20]. For instance, YTHDF1 promoted *YAP* mRNA translation by interacting with eIF3a upon depletion of m6A modification induced by ALKBH5 [39]. In addition, as described earlier, MSI2 directly binds to and effectively diminishes the translation of *SAV1* and *MOB1*, thus regulating the progression of pancreatic ductal adenocarcinoma (PDAC) [41]. Taken together, these studies indicate that RBPs could regulate the Hippo pathway through mediating target gene translation.

### Alternative splicing

RBPs could also regulate Hippo pathway via a mechanism of alternative-splicing [20]. For example, ESRP2 promotes expression of NF2 variants to activate Hippo kinases, leading to the increased activity of YAP/TAZ in chronically damaged liver [43]. Additionally, B52 regulates tissue growth by modulating Yki-specific isoforms in *Drosophila* [44]. RBFOX2 promotes the inclusion of exon 6 in *TEAD1* by directly binding to the pre-mRNA of *TEAD1*, resulting in deregulation of the Hippo pathway [45]. Together, a mechanism of alternative-splicing could be utilized by RBPs to modulate the Hippo pathway.

## Cooperation of RBPs, non-coding RNAs and transcription factors in the regulation of Hippo pathway

**Fig. 1:**
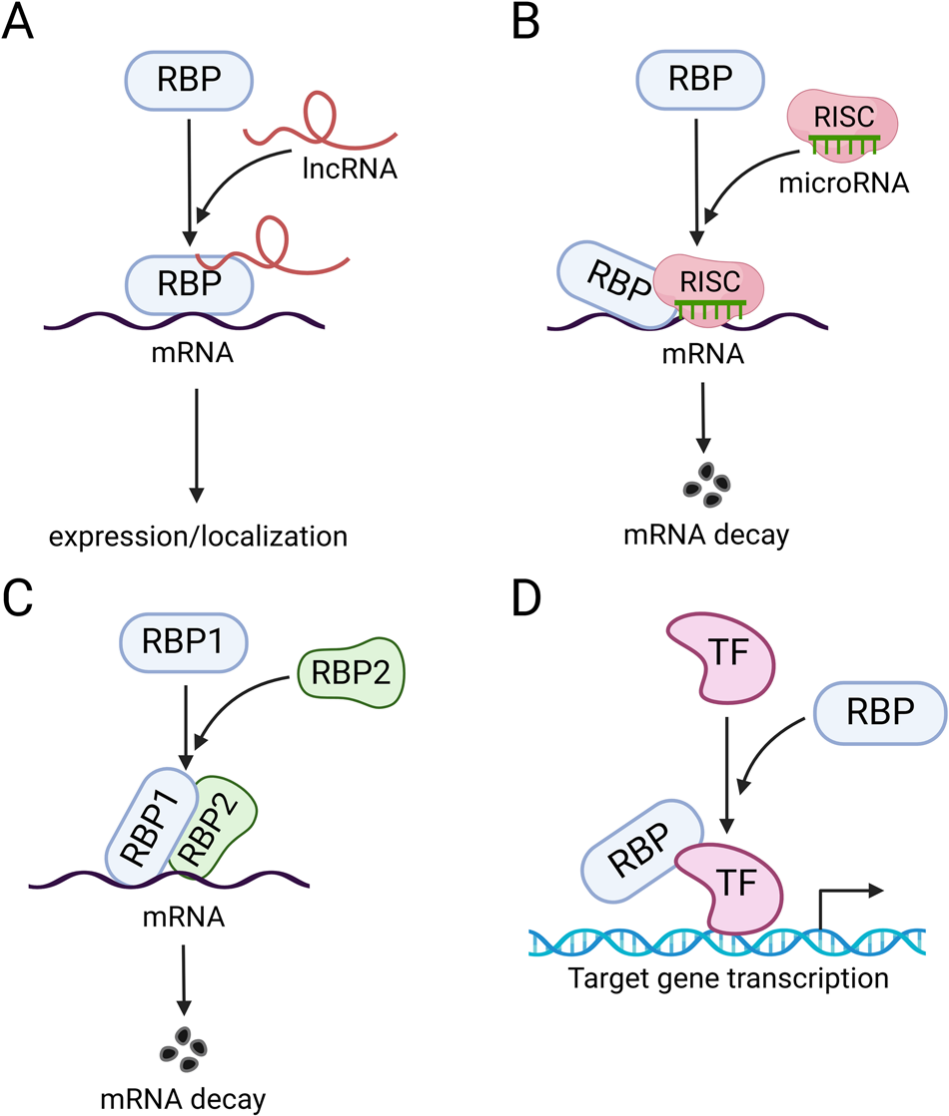
Cooperation of RBPs, non-coding RNAs, TFs in the regulation of genes expression. **A** RBP cooperates with lncRNA. **B** RBP collaborates with microRNA-RISC complex. **C** Two RBP proteins exhibit the synergistic effect. **D** RBP-TF interaction regulate gene transcription.

It’s well-documented that tight interaction between RBPs and non-coding RNAs whether cooperatively or competitively, could contribute significantly to the regulation of gene expression [20, 27, 46–49] (Fig. 1A–C). For instance, RBM47 directly interacts with lncRNA *HOXB-AS1* to disrupt the formation of *HOXB-AS1*-p53 complex, facilitating the translocation of p53 from the cytoplasm into the nucleus [50]. Besides, a series of studies have shown that RBP links lncRNA to target mRNA in multiple diseases [51, 52] (Fig. 1A). For example, lncRNA *GMDS-AS1* palliates the progression of lung adenocarcinoma (LUAD) by interacting with and recruiting TAF15 to stabilize *SIRT1* mRNA [53]. Similarly, analogous scenarios are observed within the Hippo pathway. Our previous research reveals that Rox8 recruits and stabilizes miR-8-RISC to directly bind and collaboratively trigger the degradation of *yki* mRNA in *Drosophila*, a mechanism conserved from *Drosophila* to human [19] (Fig. 1B). Of note, despites the intricate interplay between RBPs and non-coding RNAs, two RBPs may also execute synergistic effects (Fig. 1C). For instance, Lin28 recruits and cooperates with MSI2 to induce the mRNA decay of MST1/2 and LAST1/2 in TNBC [36]. In conclusion, cooperation of RBPs, non-coding RNAs and transcription factors play crucial roles in the regulation of Hippo pathway.

In addition, it has been reported that RBPs could collaborate with transcription factors (TFs) to directly bind to target DNA (Fig. 1D). For instance, RBM25 interacts with TF YY1 to bind on chromatin as a complex for altering gene transcription, as observed with targets such as NABP1, CX3CL1 and ATF7IP2 [54]. Similarly, a recent study shows that RBM39 interacts with YAP/TAZ to activate downstream gene expression, thereby contributing to resistance against indisulam [55]. Furthermore, it’s uncovered that RBPs are predominantly localized on active chromatin regions involved in transcription control [54]. On the whole, these findings suggest a general but indispensable role of RBPs in directly regulating genes transcription.

## RBPs function as downstream targets of the Hippo pathway

**Fig. 2:**
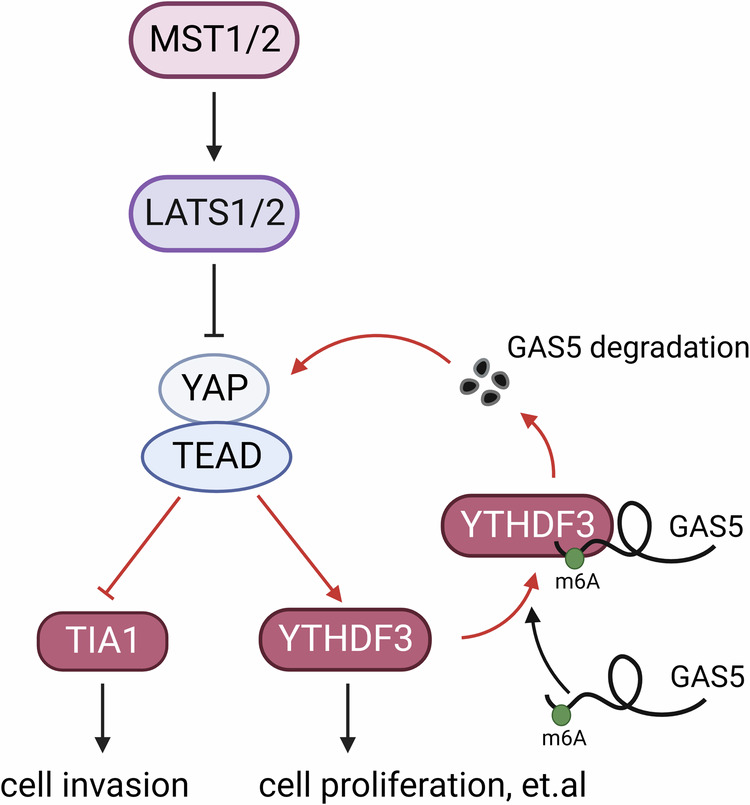
RBPs function downstream of the Hippo pathway. Expression of TIA1 and YTHDF3 regulated by Hippo pathway modulate cancer cell behaviors, like cell invasion and cell proliferation.

Extensive research has elucidated that RBPs could function as pivotal downstream targets of specific signaling pathways, orchestrating a variety of biological processes [20]. For instance, the PI3K/AKT/NF-kB signaling pathway activates *Hu-antigen R* (*HuR*) transcription in gastric cancer, promoting cellular growth and conferring resistance to apoptotic stresses [56]. Besides, RBPs serve as mediators in the crosstalk between signaling pathways [57]. For example, loss of the Hippo signaling activates JNK pathway via increasing TIA1 expression, thereby influencing cancer cell migration [14] (Fig. 2). Actually, regulatory feedback loops exemplify an intricate mechanism of interplay. For instance, YTHDF3, a downstream target of the YAP-TEAD complex, physically interacts with and induces the degradation of m6A modified-*lncRNAGAS5*. This regulation further modulates YAP phosphorylation, which is essential for its ubiquitination in colorectal cancer cells [58, 59] (Fig. 2). Hence, these studies indicate that RBPs could be involved in various biological activities through acting as downstream targets of the Hippo pathway.

## Stimuli-mediated RBP regulates the Hippo pathway

**Fig. 3:**
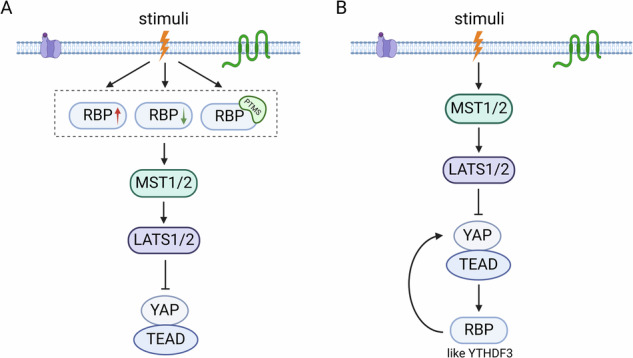
Stimuli-mediated RBPs modulate the Hippo pathway. **A** Stress alters the expression or activity of RBPs, which then regulate output of Hippo pathway. **B** A feedback regulatory loop between the Hippo pathway and RBPs.

For cellular stress adaptation, RBPs could be fine-tuned at multiple levels, including alterations in transcription activation, protein stability and binding activity through PTMs [15, 20, 60, 61] (Fig. 3A). Strikingly, RNA-binding elements within RBPs have been identified as hotspots for PTMs, representing a major mechanism underlying RBP dysfunction in diseases including cancer [20]. For example, phosphorylation of hnRNPE1 by kinase Bβ/Akt2, which is mediated by transforming growth factor-β (TGF-β), alleviates the translational repression of target transcripts encoding DAB2 and ILEI, crucial mediators of epithelial-mesenchymal transition (EMT), thereby promoting the development of breast tumors [62]. Glycogen synthase kinase 3 (GSK3) alters the conformation of RBM38 by phosphorylating it at serine 195, leading to the disruption of the interaction with the eukaryotic translation initiation factor 4E (eIF4E) on *P53* mRNA, and thus modulating P53 activity in breast and colorectal cancer cells [63].

Further more in-depth research is warranted to investigate whether specific stimuli-mediated RBPs could regulate the Hippo pathway through analogous mechanisms as described above. Actually, in a distinctive scenario, YTHDF3, a direct target of the active YAP-TEAD complex by loss of Hippo signals, being an intrinsic intracellular stressor, modulates the degradation of *lncRNAGAS5*, which is crucial for YAP ubiquitination in the cytoplasm, thus forming a feedback loop between the Hippo pathway and YTHDF3 [58] (Fig. 3B). Taken together, these data suggest RBPs may be responsive to extracellular stimuli or stresses, thereby regulating the Hippo pathway.

## Uncharacterized non-canonical RBPs to be deciphered

**Fig. 4:**
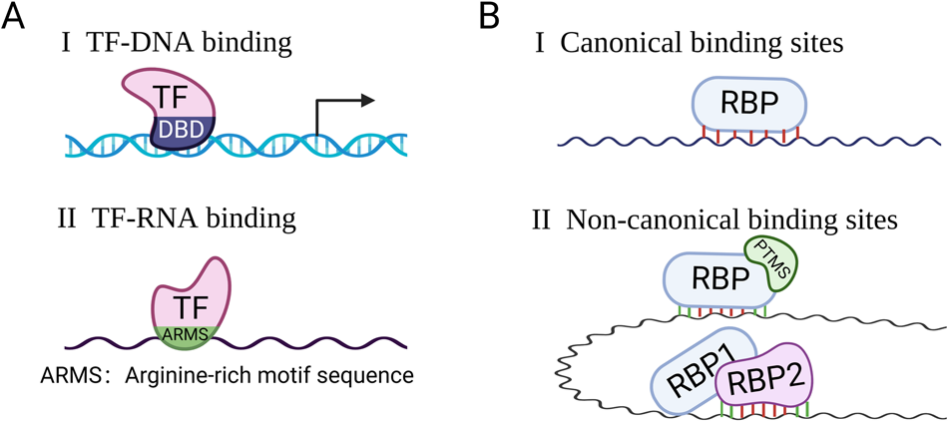
Perspective roles of RBPs in regulating the Hippo pathway. **A** TFs harbor non-canonical roles in directly binding to RNAs. **B** RBPs bind to non-canonical sites within RNA, often coupled with PTMs, RNA structure, or other proteins co-operations.

A substantial fraction of TFs function as RBPs despite lacking canonical RNA binding domains (RBDs) like RRM and KH. For instance, Estrogen receptor α (ERα), a well-known hormone receptor and pivotal driver for breast cancers through the function of being a TF, has recently been identified as a novel RBP. ERα mediates alternative splicing of *XBP1* and translation of the *eIF4G2* and *MCL1* mRNAs [61]. Of note, the RNABindRPlus database has accurately predicted the RNA-binding domain (RBD) of ERα [61, 64]. Strikingly, it has been speculated that at least half of TFs harbor a conserved RNA binding domain referred to as TF-ARMs (Arginine-rich motif sequence in TFs) [65] (Fig. 4A). Furthermore, a number of RBPs do not rely on classical RBDs for RNA binding [66], making the bioinformatic prediction of potential RBP–RNA interactions challenging. In addition, RBPs exhibit distinct affinities and preferences for core motifs in the RNA sequences, often coupled with varying flanking bases outside the core sequence, which may be due to the modulation by PTMs, interacting co-partners and local sequence or structure context within the RNA [67] (Fig. 4B). In essence, to identify potential non-canonical RBPs will undoubtedly contribute to a better understanding of crucial signaling-related development and diseases.

## Conclusion

On the whole, the review not only summarizes recent advances in the regulatory interplay between the Hippo pathway and RBPs, but expand a better understanding of potentially mechanistic regulation between RBPs and the Hippo pathway. Besides, the review also underscores the fine-tuning interaction between RBPs and non-coding RNAs in the regulation of gene expression. Moreover, we also highlight the necessity of non-canonical RBPs to be identified in future. In a word, the review may contribute to more cutting-edge research discoveries of RBPs regulating crucial signaling cascades like the Hippo pathway in the normal development and various diseases.

## Data Availability

All data relevant to this review are included in the references, tables and figures.
